# Hydrogen bonding in 1-carb­oxy­propanaminium nitrate

**DOI:** 10.1107/S1600536812013682

**Published:** 2012-04-04

**Authors:** Amel Messai, Rim Benali-Cherif, Erwann Jeanneau, Nourredine Benali-Cherif

**Affiliations:** a Laboratoire des Structures, Propriétés et Interactions Interatomiques, Université Abbes Laghrour Khenchela, 40000 Khenchela, Algeria; b Université Claude Bernard Lyon 1, Laboratoire des Multimatériaux et Interfaces (UMR 5615), 69622 Villeurbanne Cedex, France

## Abstract

There are two crystallographically independent cations and two anions in the asymmetric unit of the title compound, C_4_H_5_NO_2_^+^·NO_3_^−^. In the crystal, the 1-carb­oxy­propanaminium cations and nitrate anions are linked to each other through strong N—H⋯O and O—H⋯O hydrogen bonds, forming a three-dimensional complex network. C—H⋯O inter­actions also occur.

## Related literature

For background to inorganic–organic hybrid materials, see: Benali-Cherif, Allouche *et al.* (2007 ▶); Benali-Cherif, Kateb *et al.* (2007 ▶); Messai *et al.* (2009 ▶); Cherouana *et al.* (2003 ▶). Changes in illuminated volume were kept to a minimum, and were taken into account (Görbitz, 1999 ▶) by multi-scan inter-frame scaling.
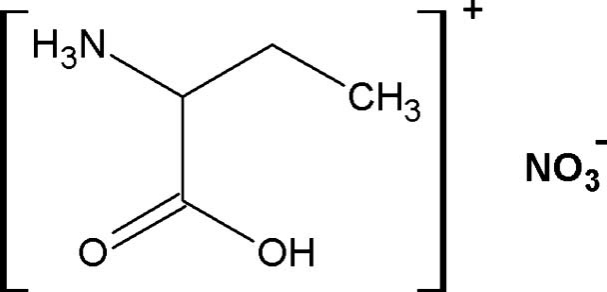


## Experimental

### 

#### Crystal data

C_4_H_10_NO_2_^+^·NO_3_^−^
*M* *_r_* = 166.14
Monoclinic, 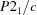

*a* = 18.274 (2) Å
*b* = 5.6052 (4) Å
*c* = 16.536 (2) Å
β = 116.224 (16)°
*V* = 1519.4 (3) Å^3^
*Z* = 8
Cu *K*α radiation
μ = 1.18 mm^−1^
*T* = 150 K
0.1 × 0.02 × 0.01 mm

#### Data collection

Oxford Xcalibur Atlas Gemini ultra diffractometer
Absorption correction: analytical (*CrysAlis PRO*; Oxford Diffraction, 2010 ▶) *T*_min_ = 0.987, *T*_max_ = 0.999
14871 measured reflections
2683 independent reflections
2441 reflections with *I* > 2σ(*I*)
*R*_int_ = 0.054

#### Refinement

*R*[*F* ^2^ > 2σ(*F* ^2^)] = 0.039
*wR*(*F* ^2^) = 0.109
*S* = 1.08
2683 reflections
203 parameters
H-atom parameters not refined
Δρ_max_ = 0.25 e Å^−3^
Δρ_min_ = −0.31 e Å^−3^

### 

Data collection: *Gemini User Manual* (Oxford Diffraction, 2006 ▶); cell refinement: *CrysAlis PRO* (Oxford Diffraction, 2010 ▶); data reduction: *CrysAlis PRO*; program(s) used to solve structure: *SIR2004* (Burla *et al.*, 2005 ▶); program(s) used to refine structure: *SHELXL97* (Sheldrick, 2008 ▶); molecular graphics: *ORTEP-3* (Farrugia, 1997 ▶) and *PLATON* (Spek, 2009 ▶); software used to prepare material for publication: *WinGX* (Farrugia, 1999 ▶).

## Supplementary Material

Crystal structure: contains datablock(s) global, I. DOI: 10.1107/S1600536812013682/ru2030sup1.cif

Structure factors: contains datablock(s) I. DOI: 10.1107/S1600536812013682/ru2030Isup2.hkl

Supplementary material file. DOI: 10.1107/S1600536812013682/ru2030Isup3.cml

Additional supplementary materials:  crystallographic information; 3D view; checkCIF report

## Figures and Tables

**Table 1:** Hydrogen-bond geometry (Å, °)

*D*—H⋯*A*	*D*—H	H⋯*A*	*D*⋯*A*	*D*—H⋯*A*
N1*A*—H1*A*⋯O1*A*^i^	0.89	2.11	2.8590 (18)	141
N1*A*—H1*A*⋯O5*B*^ii^	0.89	2.48	2.9464 (18)	113
N1*A*—H1*B*⋯O3*B*^iii^	0.89	2.01	2.8877 (17)	169
N1*A*—H1*B*⋯O4*B*^iii^	0.89	2.44	3.0033 (16)	121
N1*A*—H1*C*⋯O4*B*	0.89	1.93	2.8162 (16)	173
O2*A*—H2*O*⋯O3*B*^iv^	0.82	1.84	2.6295 (17)	160
N1*B*—H3*C*⋯O1*B*^v^	0.89	2.08	2.8470 (16)	143
N1*B*—H3*C*⋯O5*A*^v^	0.89	2.50	2.946 (2)	111
N1*B*—H3*D*⋯O3*A*^vi^	0.89	2.47	2.9917 (16)	118
N1*B*—H3*D*⋯O4*A*^vi^	0.89	2.02	2.9025 (16)	169
N1*B*—H3*E*⋯O3*A*^vii^	0.89	1.94	2.8126 (16)	168
O2*B*—H4⋯O4*A*	0.82	1.84	2.6206 (16)	159
C4*A*—H4*B*⋯O3*B*^iii^	0.96	2.58	3.382 (2)	141
C2*B*—H6⋯O3*A*^vi^	0.98	2.57	3.189 (2)	121

Symmetry codes: (i) 
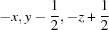
; (ii) 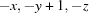; (iii) 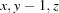; (iv) 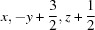; (v) 
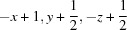
; (vi) 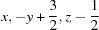; (vii) 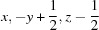.

**Fig. 1.**
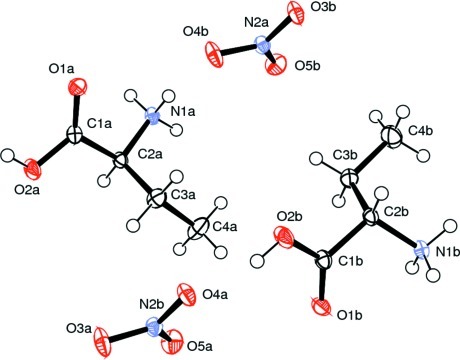
Aview of (I), showing the atomic labelling scheme and with displacement ellipsoids drawn at the 50% probability level.

**Fig. 2.**
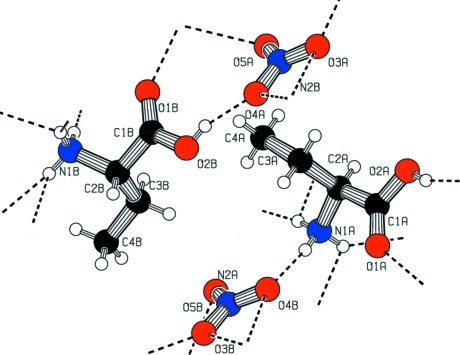
packing view of the hydrogen-bonding network

## supplementary crystallographic information

### Comment

Inorganic organic hybrid materials have been studied extensively because the blending of organic and inorganic components allows to the development of materials with novel properties. (Cherouana *et al.*, 2003; Benali-Cherif, Allouche *et al.*, 2007; Benali-Cherif, Kateb *et al.*, 2007; Messai *et al.*, 2009; In particular the family of material which combine nitrate anions with organic molecules such as aromatic and aliphatic aminoacids has been studied intensively due to their numerous uses in various fields such as biomolecular science, liquid crystals, catalysts and fuel cells.

As a contribution to the study of thiscompound family, we report in this work the synthesis and the crystal structure of a new organic cation nitrate (C_4_H_5_O_2_N)^+^(N O_3_)^-^ (I). The asymmetric unit in the structure of (I) contains two nitrate anions and two crystallographically independent monoprotonated 2-Ammonium butyric acid cations (Fig. 1). wich one one of these cations is *R* configuration and the second was the S configration. In the nitrate anions, two of the N_O distances, involving atoms O3 and O4, are slightly longer than the third, involving atom O5, while the O_N···O angles range from 117.6 (3) to 121.7 (3). The bond distances and angles of 2-Ammonium butyric acid cation are normal. The nitrate anion in (I) plays an important role in hydrogen bonding, with all three O atoms (O3, O4 and O5) being involved. The 2-Ammonium butyric acid residue forms three strong O_H··· O hydrogen bonds with the nitrate anion. The amino N atom of the phenylglycinium residue forms eight N_H···O hydrogen bonds *via* atoms O1, O2 and O3 of the nitrate anion, and two *via* the carboxyl atom O4 (Fig. 2).

### Experimental

colorlesse single crystals of this compound were obtained after a few days by slow evaporation, at room temperature, of an equimolar aqueous solution of 2-amino butyric acid and nitric acid.

### Refinement

In the absence of significant anomalous scattering, Friedel pairs were merged.

The absolute configuration was arbitrarily assigned.

The relatively large ratio of minimum to maximum corrections applied in the multiscan process (1:nnn) reflect changes in the illuminated volume of the crystal.

Changes in illuminated volume were kept to a minimum, and were taken into account (Görbitz, 1999) by the multi-scan inter-frame scaling.

All H atoms were positioned geometrically and refined with a riding model, fixing the bond lengths at 0.93 and 0.96 A ° for CH and CH3 groups, respectively. The *U*_iso_(H) values were constrained to be 1.2Ueq (parent) or 1.5Ueq (methyl C).

### Crystal data

**Table:**

C_4_H_10_NO_2_^+^·NO_3_^−^	*F*(000) = 704
*M**_r_* = 166.14	*D*_x_ = 1.453 Mg m^−^^3^
Monoclinic, *P*2_1_/*c*	Cu *K*α radiation, λ = 1.54180 Å
Hall symbol: -P 2ybc	Cell parameters from 7884 reflections
*a* = 18.274 (2) Å	θ = 4.8–66.5°
*b* = 5.6052 (4) Å	µ = 1.18 mm^−^^1^
*c* = 16.536 (2) Å	*T* = 150 K
β = 116.224 (16)°	Needle, colorless
*V* = 1519.4 (3) Å^3^	0.1 × 0.02 × 0.01 mm
*Z* = 8	

### Data collection

**Table:**

Oxford Xcalibur Atlas Gemini ultra diffractometer	2683 independent reflections
Radiation source: fine-focus sealed tube	2441 reflections with *I* > 2σ(*I*)
Graphite monochromator	*R*_int_ = 0.054
Detector resolution: 10.4685 pixels mm^-1^	θ_max_ = 66.6°, θ_min_ = 5.4°
ω scans	*h* = −21→21
Absorption correction: analytical (*CrysAlis PRO*; Oxford Diffraction, 2010)	*k* = −6→6
*T*_min_ = 0.987, *T*_max_ = 0.999	*l* = −19→19
14871 measured reflections	

### Refinement

**Table:**

Refinement on *F*^2^	Primary atom site location: structure-invariant direct methods
Least-squares matrix: full	Secondary atom site location: difference Fourier map
*R*[*F*^2^ > 2σ(*F*^2^)] = 0.039	Hydrogen site location: inferred from neighbouring sites
*wR*(*F*^2^) = 0.109	H-atom parameters not refined
*S* = 1.08	*w* = 1/[σ^2^(*F*_o_^2^) + (0.0631*P*)^2^ + 0.5656*P*] where *P* = (*F*_o_^2^ + 2*F*_c_^2^)/3
2683 reflections	(Δ/σ)_max_ < 0.001
203 parameters	Δρ_max_ = 0.25 e Å^−^^3^
0 restraints	Δρ_min_ = −0.31 e Å^−^^3^

### Special details

**Table:**

Geometry. All e.s.d.'s (except the e.s.d. in the dihedral angle between two l.s. planes) are estimated using the full covariance matrix. The cell e.s.d.'s are taken into account individually in the estimation of e.s.d.'s in distances, angles and torsion angles; correlations between e.s.d.'s in cell parameters are only used when they are defined by crystal symmetry. An approximate (isotropic) treatment of cell e.s.d.'s is used for estimating e.s.d.'s involving l.s. planes.
Refinement. Refinement of *F*^2^ against ALL reflections. The weighted *R*-factor *wR* and goodness of fit *S* are based on *F*^2^, conventional *R*-factors *R* are based on *F*, with *F* set to zero for negative *F*^2^. The threshold expression of *F*^2^ > σ(*F*^2^) is used only for calculating *R*-factors(gt) *etc*. and is not relevant to the choice of reflections for refinement. *R*-factors based on *F*^2^ are statistically about twice as large as those based on *F*, and *R*- factors based on ALL data will be even larger.

### Fractional atomic coordinates and isotropic or equivalent isotropic displacement parameters (Å^2^)

**Table:**

	*x*	*y*	*z*	*U*_iso_*/*U*_eq_	
O1A	0.05317 (6)	0.57372 (18)	0.29774 (7)	0.0216 (3)	
O2A	0.11711 (7)	0.2856 (2)	0.39898 (7)	0.0261 (3)	
H2O	0.1042	0.3668	0.4319	0.039*	
N1A	0.06545 (7)	0.3190 (2)	0.16452 (8)	0.0153 (3)	
H1A	0.0142	0.2831	0.1526	0.023*	
H1B	0.0799	0.2372	0.1277	0.023*	
H1C	0.0693	0.4746	0.1563	0.023*	
C1A	0.09177 (9)	0.3901 (3)	0.32014 (10)	0.0163 (3)	
C2A	0.12050 (8)	0.2556 (2)	0.25965 (9)	0.0152 (3)	
H2	0.1166	0.0838	0.2680	0.018*	
C3A	0.20896 (9)	0.3193 (3)	0.28412 (11)	0.0216 (3)	
H3A	0.2119	0.4876	0.2722	0.026*	
H3B	0.2415	0.2939	0.3482	0.026*	
C4A	0.24575 (10)	0.1760 (3)	0.23296 (12)	0.0299 (4)	
H4A	0.3012	0.2252	0.2515	0.045*	
H4B	0.2148	0.2030	0.1694	0.045*	
H4C	0.2445	0.0093	0.2456	0.045*	
O1B	0.45105 (7)	0.41637 (18)	0.24898 (7)	0.0221 (3)	
O2B	0.37874 (8)	0.6976 (2)	0.27877 (8)	0.0294 (3)	
H4	0.3942	0.6267	0.3269	0.044*	
N1B	0.43613 (7)	0.6551 (2)	0.09954 (8)	0.0154 (3)	
H3C	0.4859	0.7012	0.1386	0.023*	
H3D	0.4215	0.7326	0.0477	0.023*	
H3E	0.4362	0.4988	0.0900	0.023*	
C1B	0.40800 (9)	0.5918 (3)	0.22859 (10)	0.0173 (3)	
C2B	0.37732 (9)	0.7096 (2)	0.13665 (9)	0.0163 (3)	
H6	0.3747	0.8827	0.1434	0.020*	
C3B	0.29190 (9)	0.6152 (3)	0.07476 (10)	0.0243 (4)	
H7A	0.2946	0.4431	0.0704	0.029*	
H7B	0.2557	0.6503	0.1019	0.029*	
C4B	0.25584 (10)	0.7202 (4)	−0.01973 (11)	0.0341 (4)	
H8A	0.2026	0.6538	−0.0548	0.051*	
H8B	0.2906	0.6829	−0.0478	0.051*	
H8C	0.2516	0.8902	−0.0163	0.051*	
O4B	0.08435 (8)	0.80167 (19)	0.13033 (7)	0.0262 (3)	
O3B	0.08960 (7)	1.04237 (18)	0.03095 (7)	0.0226 (3)	
N2A	0.08566 (7)	0.8294 (2)	0.05545 (8)	0.0170 (3)	
O3A	0.41482 (8)	0.32834 (19)	0.54878 (8)	0.0275 (3)	
O4A	0.41008 (7)	0.56070 (17)	0.44238 (7)	0.0212 (3)	
O5A	0.41568 (7)	0.17533 (18)	0.42863 (8)	0.0246 (3)	
N2B	0.41346 (7)	0.3502 (2)	0.47249 (8)	0.0173 (3)	
O5B	0.08332 (7)	0.65860 (18)	0.00782 (8)	0.0248 (3)	

### Atomic displacement parameters (Å^2^)

**Table:**

	*U*^11^	*U*^22^	*U*^33^	*U*^12^	*U*^13^	*U*^23^
O1A	0.0271 (6)	0.0212 (6)	0.0200 (6)	0.0077 (4)	0.0136 (5)	0.0009 (4)
O2A	0.0408 (7)	0.0267 (6)	0.0164 (6)	0.0118 (5)	0.0178 (5)	0.0036 (4)
N1A	0.0201 (6)	0.0136 (6)	0.0140 (6)	−0.0013 (4)	0.0092 (5)	−0.0015 (4)
C1A	0.0190 (7)	0.0164 (7)	0.0154 (7)	−0.0021 (5)	0.0093 (6)	−0.0007 (5)
C2A	0.0193 (7)	0.0140 (6)	0.0134 (7)	0.0017 (5)	0.0082 (6)	0.0002 (5)
C3A	0.0195 (8)	0.0246 (8)	0.0201 (8)	0.0000 (6)	0.0083 (6)	−0.0039 (6)
C4A	0.0227 (8)	0.0391 (10)	0.0325 (9)	−0.0003 (7)	0.0163 (7)	−0.0078 (7)
O1B	0.0280 (6)	0.0220 (6)	0.0182 (5)	0.0087 (4)	0.0119 (5)	0.0064 (4)
O2B	0.0450 (7)	0.0321 (6)	0.0190 (6)	0.0178 (5)	0.0213 (6)	0.0091 (5)
N1B	0.0197 (6)	0.0141 (6)	0.0141 (6)	−0.0016 (4)	0.0091 (5)	0.0000 (4)
C1B	0.0215 (7)	0.0164 (7)	0.0156 (7)	−0.0004 (5)	0.0096 (6)	−0.0005 (5)
C2B	0.0210 (7)	0.0157 (7)	0.0153 (7)	0.0030 (5)	0.0110 (6)	0.0021 (5)
C3B	0.0191 (8)	0.0329 (8)	0.0212 (8)	0.0008 (6)	0.0092 (7)	0.0065 (6)
C4B	0.0230 (9)	0.0497 (11)	0.0232 (9)	−0.0038 (8)	0.0043 (7)	0.0112 (8)
O4B	0.0474 (7)	0.0192 (5)	0.0168 (6)	−0.0034 (5)	0.0184 (5)	0.0011 (4)
O3B	0.0373 (6)	0.0165 (5)	0.0196 (6)	−0.0017 (4)	0.0176 (5)	0.0019 (4)
N2A	0.0198 (6)	0.0173 (6)	0.0143 (6)	−0.0006 (5)	0.0079 (5)	−0.0013 (5)
O3A	0.0522 (8)	0.0186 (5)	0.0228 (6)	0.0024 (5)	0.0268 (6)	0.0028 (4)
O4A	0.0334 (6)	0.0158 (5)	0.0176 (5)	0.0026 (4)	0.0140 (5)	0.0034 (4)
O5A	0.0320 (6)	0.0208 (5)	0.0242 (6)	−0.0005 (4)	0.0153 (5)	−0.0083 (4)
N2B	0.0199 (6)	0.0172 (6)	0.0168 (6)	0.0005 (5)	0.0099 (5)	−0.0002 (5)
O5B	0.0321 (6)	0.0208 (6)	0.0235 (6)	0.0007 (4)	0.0141 (5)	−0.0079 (4)

### Geometric parameters (Å, º)

**Table:**

O1A—C1A	1.2098 (18)	N1B—C2B	1.4859 (17)
O2A—C1A	1.3127 (18)	N1B—H3C	0.8900
O2A—H2O	0.8200	N1B—H3D	0.8900
N1A—C2A	1.4880 (18)	N1B—H3E	0.8900
N1A—H1A	0.8900	C1B—C2B	1.520 (2)
N1A—H1B	0.8900	C2B—C3B	1.534 (2)
N1A—H1C	0.8900	C2B—H6	0.9800
C1A—C2A	1.5193 (19)	C3B—C4B	1.521 (2)
C2A—C3A	1.525 (2)	C3B—H7A	0.9700
C2A—H2	0.9800	C3B—H7B	0.9700
C3A—C4A	1.522 (2)	C4B—H8A	0.9600
C3A—H3A	0.9700	C4B—H8B	0.9600
C3A—H3B	0.9700	C4B—H8C	0.9600
C4A—H4A	0.9600	O4B—N2A	1.2586 (16)
C4A—H4B	0.9600	O3B—N2A	1.2727 (16)
C4A—H4C	0.9600	N2A—O5B	1.2285 (16)
O1B—C1B	1.2103 (18)	O3A—N2B	1.2568 (16)
O2B—C1B	1.3108 (18)	O4A—N2B	1.2714 (16)
O2B—H4	0.8200	O5A—N2B	1.2307 (16)
			
C1A—O2A—H2O	109.5	H3C—N1B—H3D	109.5
C2A—N1A—H1A	109.5	C2B—N1B—H3E	109.5
C2A—N1A—H1B	109.5	H3C—N1B—H3E	109.5
H1A—N1A—H1B	109.5	H3D—N1B—H3E	109.5
C2A—N1A—H1C	109.5	O1B—C1B—O2B	126.01 (13)
H1A—N1A—H1C	109.5	O1B—C1B—C2B	122.62 (13)
H1B—N1A—H1C	109.5	O2B—C1B—C2B	111.30 (12)
O1A—C1A—O2A	125.85 (13)	N1B—C2B—C1B	108.01 (11)
O1A—C1A—C2A	123.02 (13)	N1B—C2B—C3B	111.22 (12)
O2A—C1A—C2A	111.08 (12)	C1B—C2B—C3B	109.52 (12)
N1A—C2A—C1A	107.98 (11)	N1B—C2B—H6	109.4
N1A—C2A—C3A	111.51 (11)	C1B—C2B—H6	109.4
C1A—C2A—C3A	110.08 (12)	C3B—C2B—H6	109.4
N1A—C2A—H2	109.1	C4B—C3B—C2B	113.77 (13)
C1A—C2A—H2	109.1	C4B—C3B—H7A	108.8
C3A—C2A—H2	109.1	C2B—C3B—H7A	108.8
C4A—C3A—C2A	113.94 (13)	C4B—C3B—H7B	108.8
C4A—C3A—H3A	108.8	C2B—C3B—H7B	108.8
C2A—C3A—H3A	108.8	H7A—C3B—H7B	107.7
C4A—C3A—H3B	108.8	C3B—C4B—H8A	109.5
C2A—C3A—H3B	108.8	C3B—C4B—H8B	109.5
H3A—C3A—H3B	107.7	H8A—C4B—H8B	109.5
C3A—C4A—H4A	109.5	C3B—C4B—H8C	109.5
C3A—C4A—H4B	109.5	H8A—C4B—H8C	109.5
H4A—C4A—H4B	109.5	H8B—C4B—H8C	109.5
C3A—C4A—H4C	109.5	O5B—N2A—O4B	121.59 (12)
H4A—C4A—H4C	109.5	O5B—N2A—O3B	121.18 (11)
H4B—C4A—H4C	109.5	O4B—N2A—O3B	117.22 (11)
C1B—O2B—H4	109.5	O5A—N2B—O3A	121.51 (12)
C2B—N1B—H3C	109.5	O5A—N2B—O4A	121.13 (12)
C2B—N1B—H3D	109.5	O3A—N2B—O4A	117.36 (11)
			
O1A—C1A—C2A—N1A	−24.57 (18)	O1B—C1B—C2B—N1B	−26.22 (19)
O2A—C1A—C2A—N1A	157.91 (12)	O2B—C1B—C2B—N1B	156.73 (12)
O1A—C1A—C2A—C3A	97.38 (16)	O1B—C1B—C2B—C3B	95.05 (16)
O2A—C1A—C2A—C3A	−80.14 (15)	O2B—C1B—C2B—C3B	−82.00 (15)
N1A—C2A—C3A—C4A	−65.55 (16)	N1B—C2B—C3B—C4B	−60.01 (17)
C1A—C2A—C3A—C4A	174.63 (13)	C1B—C2B—C3B—C4B	−179.33 (13)

### Hydrogen-bond geometry (Å, º)

**Table:**

*D*—H···*A*	*D*—H	H···*A*	*D*···*A*	*D*—H···*A*
N1*A*—H1*A*···O1*A*^i^	0.89	2.11	2.8590 (18)	141
N1*A*—H1*A*···O5*B*^ii^	0.89	2.48	2.9464 (18)	113
N1*A*—H1*B*···O3*B*^iii^	0.89	2.01	2.8877 (17)	169
N1*A*—H1*B*···O4*B*^iii^	0.89	2.44	3.0033 (16)	121
N1*A*—H1*C*···O4*B*	0.89	1.93	2.8162 (16)	173
O2*A*—H2*O*···O3*B*^iv^	0.82	1.84	2.6295 (17)	160
N1*B*—H3*C*···O1*B*^v^	0.89	2.08	2.8470 (16)	143
N1*B*—H3*C*···O5*A*^v^	0.89	2.50	2.946 (2)	111
N1*B*—H3*D*···O3*A*^vi^	0.89	2.47	2.9917 (16)	118
N1*B*—H3*D*···O4*A*^vi^	0.89	2.02	2.9025 (16)	169
N1*B*—H3*E*···O3*A*^vii^	0.89	1.94	2.8126 (16)	168
O2*B*—H4···O4*A*	0.82	1.84	2.6206 (16)	159
C4*A*—H4*B*···O3*B*^iii^	0.96	2.58	3.382 (2)	141
C2*B*—H6···O3*A*^vi^	0.98	2.57	3.189 (2)	121

Symmetry codes: (i) −*x*, *y*−1/2, −*z*+1/2; (ii) −*x*, −*y*+1, −*z*; (iii) *x*, *y*−1, *z*; (iv) *x*, −*y*+3/2, *z*+1/2; (v) −*x*+1, *y*+1/2, −*z*+1/2; (vi) *x*, −*y*+3/2, *z*−1/2; (vii) *x*, −*y*+1/2, *z*−1/2.
